# The Interplay Between Absolute Language and Moral Reasoning on Endorsement of Moral Foundations

**DOI:** 10.3389/fpsyg.2021.569380

**Published:** 2021-04-30

**Authors:** Kevin L. Blankenship, Traci Y. Craig, Marielle G. Machacek

**Affiliations:** ^1^Department of Psychology, Iowa State University, Ames, IA, United States; ^2^Department of Psychology and Communication, University of Idaho, Moscow, ID, United States

**Keywords:** moral reasoning, individual differences, language, morality, deontological

## Abstract

Morality – the subjective sense that humans discern between right and wrong – plays a ubiquitous role in everyday life. Deontological reasoning conceptualizes moral decision-making as rigid, such that many moral choices are forbidden or required. Not surprisingly, the language used in measures of deontological reasoning tends to be rigid, including phrases such as “always” and “never.” Two studies (*N* = 553) drawn from two different populations used commonly used measures of moral reasoning and measures of morality to examine the link between individual differences in deontological reasoning and language on the endorsement of moral foundations. Participants low on deontological reasoning generally showed less endorsement for moral principles when extreme language was used in the measures (relative to less extreme language).

## Introduction

Much like many social phenomena, language is the primary mechanism through which moral decisions are conceptualized and conveyed to others. For example, stories in many cultures are used to teach what is morally correct (Bible, Chinese proverbs, etc.). It is no surprise, then, that language is intertwined with moral reasoning and is part of an individual's moral architecture (Costa et al., 2014).

Philosophers have outlined two general reasoning styles that lead to different responses to moral decisions. A deontological mindset is one where a “hardline” approach is implemented, such that there is no moral flexibility (e.g., under no conditions is killing someone acceptable; (Kant, 1785/1959). On the other hand, a consequentialist mindset acknowledges that, when harm benefits the greater good, it is acceptable to engage in a seemingly immoral action (Mill, 1861).

Examination of common individual difference measures of deontological reasoning provide numerous examples of rigid or absolute language – phrases, when paired with beliefs and actions imply the “correct” response and even presents the actions as dichotomous. For example, the term “never” is used many items of the Consequentialist Thinking scale (Robinson et al., 2015) and Ethical Position scale (Forsyth, 1980). Given the conceptual meaning of deontological reasoning, it is no surprise that such a rigid or extreme language style would be included in measures of a rather rigid reasoning (see also (Giammarco, 2016). Perhaps it is the language used in these items that leads respondents low in deontological reasoning to indicate low levels of agreement with these items. Alternatively, the low levels of agreement may be driven more by the extreme language rather than by the content or meaning of the item itself.

Notably, individuals who score low on scales of moral rigidity do not necessarily score high on other measures of moral reasoning, such as utilitarianism (*r* = −0.14; (Robinson et al., 2015) and relativism (*r* = 0.05; Forsyth, 1980), suggesting that the deontological and consequentialist reasoning styles are not unidimensional. Thus, it is possible that rejection of the deontological items expressed via low scores on deontological reasoning do not necessarily mean endorsement of other types of moral reasoning. It may simply mean, because of the connection between the reasoning style and extreme language, they reject the rigid language style used in the items.

We argue that individuals low in deontological reasoning would be more sensitive to the inclusion of extreme language in statements of morality than individuals high in deontological reasoning and thus be less likely to agree with the statements. Some indirect support for this comes from the research on language styles in the persuasion literature. Rigid and absolute language can modify the intended assertion in a communicative act (Holtgraves, 2002) and yield less agreement with topics when messages contain this type of language (Blankenship and Craig, 2011, Craig and Blankenship, 2011). Use of similar language styles in other measures has been shown to affect agreement in questionnaires as well. For example, individuals were less likely to agree with statements about a previous behavior when the statements included more extreme phrases such as “frequently” than less extreme phrases such as “occasionally” (Salancik and Conway, 1975). The extreme language modifies the extremity of the statements and therefore people are less likely to fully endorse statements containing “frequently” than they are to the same statements containing “occasionally,” in part because it is easier to generate instances of a behavior one has occasionally engaged in rather than frequently (Schwarz et al., 1991).

In addition, Social Judgment theory (Sherif and Hovland, 1961) maintains that people compare information they are confronted with to their opinions. Agreement with that information depends on whether it falls within one of three latitudes or ranges. Information falling within one's latitude of acceptance (a range of positions that one finds acceptable) will lead to endorsement of that statement. On the other hand, statements falling within one's latitude of rejection (a range of positions that one will not accept) will lead to rejection of that statement. Thus, a social judgment theory perspective would suggest that, compared to non-extreme statements, statements containing extreme language may fall into one's latitude of rejection, resulting in reduced endorsement.

Extending this understanding of extreme language to measures of moral judgments, items using extreme or rigid statements may make salient the absolute nature of the moral statements and alter their connotation to be more extreme People may therefore respond to morality-relevant items based more on the modifier language (*always/never*) rather than the morality of the act that is the focus of the statement itself. When considering how morality is currently measured, it is also important to understand the role of language markers in deontological reasoning. People high in deontological reasoning view the world in absolutes and may find these extreme language markers to reflect their sincerely held beliefs. However, for individuals low on deontological reasoning, they may resist endorsing statements that use extreme or absolute language and therefore not endorse items that would otherwise fit well with their moral beliefs. Thus, deontological reasoning may qualify the extent to which measures of moral reasoning are influenced by extreme language markers.

## Research Overview

Previous research has demonstrated that absolute language can decrease agreement with statements about social and economic issues (e.g., Salancik and Conway, 1975). However, little research has examined this effect on a topic or in a context where absolute language is closely linked to moral reasoning. These hypotheses, while consistent with previous research on language styles and attitudes (Holtgraves, 2010), go beyond previous research to posit that absolute language is more intricately tied to moral reasoning than to other topics where similar language manipulations have been used (e.g., being environmentally friendly; Chaiken and Yates, 1981; church behaviors; Salancik and Conway, 1975; personality measures; (Petrocelli et al., 2010). Thus, rigid language and deontological reasoning seem to go together, unlike conceptualizing being environmentally friendly, for example. Individuals high in deontological reasoning report agreement with statements such as “*Some rules should never be broken*” (Robinson et al., 2015) and “*It is never necessary to sacrifice the welfare of others*” (Forsyth, 1980) because that is an accurate reflection of the world to them.

Similarly, moral judgments seem to be qualitatively different from other judgments (Bartels et al., 2014), in part because they relate to relatively universal standards of conduct that cut across cultures and generations (Machery and Mallon, 2010). Individuals are often intolerant of differences based in morality (e.g., incest), but not other standards (e.g., formal greetings). It is no surprise, then, that statements about morally “correct” decisions and actions (Skitka et al., 2005) would include such absolute and rigid language. And yet for individuals low in deontological reasoning, the linguistic rigidity in these measures provides few opportunities for them to endorse items that reflect their own moral reasoning.

To recap, we believe that individuals low in deontological reasoning would be more sensitive to the inclusion of extreme language in statements of morality than individuals high in deontological reasoning. We chose to test these hypotheses with responses to the Moral Foundations Questionnaire (MFQ; Graham et al., 2011) as the outcome of interest. The MFQ consists of two parts; individuals first report whether the foundations are relevant to their moral decision making (e.g., whether someone acted unfairly is relevant to their moral decisions). Individuals then report their agreement with a number of statements relevant to the moral foundation (e.g., justice is an important requirement for a society). Because we are interested in how extreme language affects endorsement of moral statements, the latter endorsement portion of the MFQ is most relevant to the present research.

Moreover, the MFQ has been used in numerous studies and the endorsement portion of the measure contains little to no absolute language in the items – unlike commonly used measures of moral reasoning described earlier. Thus, we decided that the MFQ would provide an ideal testbed for the linguistic manipulation. That is, by creating an experimental condition whereby absolute phrases are embedded in the statements and comparing responses to statements that were relatively less rigid, we can assess whether absolute language has an influence on the endorsement of the moral foundations for those low in self-reported deontological reasoning. Across these studies, we offer new insights into the link between language style and moral reasoning, showing that language styles do matter in the conceptualization of morality.

## Pilot Testing

### Materials and Methods

An adaptation of the Endorsement items on the Moral Foundations Questionnaire (MFQ), was pilot tested to determine whether phrasing the MFQ items in either a “relative” or “absolute” frame would have an influence on item extremity, concreteness, and clarity. For example, the original MFQ item “*I think it's morally wrong that rich children inherit a lot of money while poor children inherit nothing*” was phrased “*I think it's* *always* *morally wrong that rich children inherit a lot of money while poor children inherit nothing*” in the absolute condition and “*There* *are times* *where I think it's morally wrong that rich children inherit a lot of money while poor children inherit nothing*” in the relative condition. A full list of the items in each condition can be found in Appendix A.

Seventy-eight undergraduates (54% female, 81% White/Caucasian, mean age of 19; *SD* = 1.13) were recruited from a large Midwestern university and were randomly assigned to one of two conditions, relative or absolute morality framing. In each condition, the participant first read the MFQ item and rated their agreement on a 6-point rating scale (“*Strongly Disagree*” to “*Strongly Agree*”). Next, participants were asked to rate each statement on multiple 5-point semantic rating scales for the following dimensions: *Not Extreme/Extreme, Flexible/Concrete, Uncertain/Absolute, Ambiguous/Definite*, and *Vague/Clear*.

### Results

Ratings for the *Flexible/Concrete, Uncertain/Absolute*, and *Ambiguous/Definite* dimensions were combined for each item, with internal consistency estimates ranging from 0.73 to 0.89. Ratings scores were then combined using the Individuating (i.e., Harm and Fairness) and Binding (Loyalty, Authority, and Purity) subscales of the MFQ. Results of independent samples *t*-tests revealed items with the absolute framing were rated as more extreme, more concrete, and clearer compared to those who read the relative framed items, with all effect size estimates ranging from moderate to strong. See Table 1 for full results. Having established a manipulation that affects extremity and concreteness moral statements, we used these items in a series of studies.

**Table 1:** Effects of absolute vs. relative framing on dimensions of item wording extremity, concreteness, and clarity.

**Subscale**	**Rating criteria**	**Absolute**		**Relative**		***t***	***p***	***d***
		***M***	***SD***	***M***	***SD***			
Individuating	Extreme	3.48	0.73	3.15	0.67	2.11	0.04^*^	0.47
	Concrete^∧^	3.63	0.54	3.07	0.6	4.36	0.001^**^	0.98
	Clear	3.6	0.71	2.94	0.78	3.95	0.001^**^	0.88
Binding	Extreme	3.35	0.69	2.79	0.65	3.73	0.001^**^	0.68
	Concrete^∧^	3.28	0.56	2.84	0.53	3.53	0.001^*^	0.84
	Clear	3.19	0.56	2.77	0.68	2.96	0.004^*^	0.67

* *p < 0.05*,

** *p < 0.001*,

∧ *Concrete refers to the combined rating dimensions of Flexible/Concrete, Uncertain/Absolute, and Ambiguous/Definite; Individuating Subscale encompasses Harm and Fairness foundation items; Binding Subscale encompasses Loyalty, Authority, and Purity foundation items*.

## Study 1

### Methods

#### Participants and Procedure

One hundred fifty-three participants (71 male, 74 female; *M*_age_ = 40.8, *SD*_age_ = 11.95; 118 Caucasian, 14 Black/African American, 7, Asian/Pacific Islander, 4 Hispanic, 1 East Asian, 1 Other) recruited from Amazon's Mechanical Turk (Mturk) participated in a 2 (Language: relative vs. absolute) × Reasoning style (continuous) design^1^. The study was administered through the Qualtrics platform. After consenting, participants completed two measures that assess the deontological aspects of the higher order construct of moral reasoning: the Consequentialist Thinking Style scale (CTS; Piazza and Sousa, 2014) and the Consequentialist scale (Robinson et al., 2015). Afterward, participants completed the 15-item endorsement section of the portion of the Moral Foundations Questionnaire (Part 2; Graham et al., 2011)^2^. Embedded within the items was the Language manipulation, such that in the relative condition the scale items included phrases such as “sometimes” whereas in the absolute condition the scale items included “always” (see Salancik and Conway, 1975 for a similar manipulation). After completing the measures, participants reported their political ideology and demographics, were debriefed, and paid $1.00 for participating^3^.

### Predictor Variables

#### Consequentialist Scale

Participants completed the Consequentialist Scale (Robinson et al., 2015), a 10-item measure that assesses two types of moral reasoning: utilitarian beliefs (e.g., “*Rules and laws should only be followed when they maximize happiness*”) and deontological beliefs (e.g., “*It is never morally justified to cause someone harm*”). Participants were given the items in random order and rated their agreement on a 5-point scale (1 = *completely disagree*, 5 = *completely agree*). Both the utilitarian beliefs sub-scale (α = 0.90) and deontological beliefs sub-scale (α = 0.81) demonstrated good internal reliability. Higher scores indicate greater endorsement of utilitarian and deontological beliefs.

#### Consequentialist Thinking Style Scale

Following the consequentialist scale, participants completed the Consequentialist Thinking Style scale (Piazza and Landy, 2013), whereby participants report whether 14 behaviors are morally permissible on a three-point scale (1 = *never morally permissible*, 2, = *permissible if it produces more good than bad*, 3 = *obligatory if it produces more good than bad)*. Sample behaviors include killing, assisted suicide, torture, cannibalism, and betrayal. The items were reverse scored and combined to create an overall index of reasoning style (α = 0.90). Higher scores indicate greater endorsement of deontological reasoning.

#### Political Ideology

We included a measure of political ideology for exploratory purposes. Following all dependent measures and prior to the demographic measures, participants reported their political ideology on three 9-point scales. Specifically, participants reported how liberal or conservative they are in general, in relation to economic policy, and in relation to social policy (1 = *Extremely Liberal*; 9 = *Extremely Conservative;* α = 0.94; for a similar measure, see (Nail et al., 2009). Higher scores indicate greater political conservatism.

### Independent Variables

#### Linguistic Manipulation

All participants completed the agreement portion of the MFQ (Part 2), which was adapted for this study. Specifically, participants in the relative condition were provided statements with linguistic qualifiers (e.g., sometimes), whereas participants in the absolute condition were provided statements with the qualifiers (e.g., always; see Appendix A for exact wording of items)^4^. Items were presented in random order.

### Dependent Measure

#### MFQ Scores

Because the language of the MFQ statements served as the independent variable, participants' agreement with those statements served as the dependent variable. Participants reported their agreement with those items on a 6-point scale (1 = *strongly disagree*, 6 = *strongly agree*). Consistent with Graham et al. (2011), the Harm and Fairness subscales were combined to create an index of Individuating foundations (α = 0.65), and the remaining three subscales were combined to create the Binding foundations (α = 0.79)^5^.

### Results

Table 2 presents the means, standard deviations, and correlations of the relevant variables. We expected that individual differences in self-reported deontological reasoning would interact with the language manipulation, such that the effect of the language manipulation would be more pronounced for participants low in deontological reasoning than high. Because the two scales measuring deontological reasoning were correlated (*r* = 0.43, see Table 2), participants' scores were standardized and combined to create an overall index of deontological reasoning, which served as the moderator in the analyses along with the language manipulation^6^.

**Table 2:** Study 1 means, standard deviations, and correlations of the variables.

	***M* (*SD*)**	**2**.	**3**.	**4**.	**5**.	**6**.	**7**.	**8**.	**9**.
1. INDIV	4.76 (0.74)	0.003	0.47^*^	−0.30^*^	0.34^*^	−0.14	−0.05	0.18^*^	0.18^*^
2. BIND	3.80 (0.92)	–	0.88^*^	0.46^*^	0.46^*^	0.34^*^	0.39^*^	0.05	0.02
3. MFTOT	4.19 (0.63)		–	0.27^*^	0.57^*^	0.23^*^	0.37^*^	0.13	0.10
4. PO	4.34 (2.29)			–	0.12	0.11	0.33^*^	0.06	−0.002
5. DEON	4.90 (1.25)				–	0.08	0.43^*^	0.20^*^	0.09
6. UTIL	2.81 (1.46)					–	−0.10	−0.23^*^	−0.04
7. CTS	22.56 (4.97)						–	0.02	0.04
8. AGE	40.77 (11.95)							–	0.08
9. GEN									–

* *p < 0.05. INDIV, Individualizing foundations; BIND, Binding foundations; MFTOT, Moral foundations total scale; PO, Political orientation; DEON, Deontology scale; UTIL, Utilitarian scale; CTS, Consequentialist Thinking Style scale; AGE, Participant age; GEN, Participant gender*.

#### MFQ Total Score

We submitted participants' endorsement with the MFQ items to a 2 (Language: relative vs. absolute) × Reasoning style (continuous) regression using the PROCESS macro (Model 1; Hayes, 2013). Results revealed a main effect of Reasoning style [*b* = 0.42, *SE* = 0.05, *t*(149) = 8.27, *p* < 0.001, 95% *CI*: 0.32, 0.52], with greater deontological reasoning associated with greater agreement. The predicted interaction was marginally significant (see Table 3). At 1 *SD* below the mean of deontological reasoning style scores, there was less agreement when the statements contained absolute terms (*M* = 3.67) than relative terms (*M* = 4.0). A Johnson-Neyman analysis (Johnson and Neyman, 1936) revealed that the range of significance included 48% of the sample below the mean of the reasoning style measure. However, at 1 *SD* above the mean of reasoning style scores however, the Language manipulation did not affect agreement. Overall, a deontological reasoning style moderated the influence of absolute vs. relative language on commonly used measures of morality.

**Table 3:** Study 1 interaction effects of language × reasoning style (continuous) regression on MFQ total scores, and individuating subscale.

		***b***	***SE***	***t*(149)**	***p***	**95% *CI***
MFTOT						
	Interaction	0.10	0.05	1.97	0.051	−0.0002;0.21
	Decomposed at +1 *SD* Deon. reasoning	0.004	0.06	0.07	0.94	−0.12;0.12
	Decomposed at −1 *SD* Deon. reasoning	−0.16	0.06	−2.76	0.007	−0.28;−0.04
INDIV subscale						
	Interaction	0.19	0.07	2.69	0.008	0.05;0.33
	Decomposed at +1 *SD* Deon. reasoning	0.08	0.08	0.97	0.33	−0.08;0.24
	Decomposed at −1 *SD* Deon. reasoning	−0.23	0.08	−2.87	0.005	−0.39;−0.07

*MFTOT, Moral foundations total scale; INDIV, Individualizing foundations*.

As noted earlier, Graham et al. (2011) further breaks down the MFQ into the Individualizing and Binding Subscales. The Harm and Fairness subscales comprise the Individualizing scale and the Loyalty, Authority, and Purity subscales make up the Binding scale. The subscales have been useful in examining differences in moral foundations across the ideological spectrum (Graham et al., 2013). Previous research has found that the subscales differ in their relation to each other (Piazza and Landy, 2013; see also Table 2). We therefore examined whether the subscales were differentially affected by the interplay between the language manipulation and moral reasoning. To do this, we submitted participants' agreement with the Individualizing and Binding indices to separate 2 (Language: relative vs. absolute) × Reasoning style (continuous) regressions.

#### Individualizing Subscale

Results revealed a main effect of Reasoning style [*b* = 0.2, *SE* = 0.07, *t*(149) = 2.83, *p* = 0.005, 95% *CI:* 0.06, 0.33], with greater deontological reasoning associated with greater agreement. The Language × Reasoning style interaction was also significant (see Table 3). Specifically, at 1 *SD* below the mean of the deontological reasoning style index, participants in the absolute conditions (*M* = 4.36) reported less agreement than in the relative conditions (*M* = 4.82). The range of significance included 41% of the sample below the mean of the reasoning style measure. At 1 *SD* above the mean of reasoning style scores, the Language effect was not significant. Thus, participants low on a deontological reasoning style reported less agreement on the Individualizing subscale when the items contained absolute than qualifying language.

#### Binding Subscale

Results revealed a main effect of Reasoning style [*b* = 0.57, *SE* = 0.08, *t*(149) = 7.24, *p* < 0.001, 95% *CI*: 0.41, 0.72], with higher deontological reasoning scores associated with greater agreement. Unlike the individualizing subscale, however, neither the Language effect (*p* = 0.21) nor the Language × Idealism interaction were significant (*p* = 0.6)^7^^,^^8^.

#### Political Ideology as a Moderator

Although not crucial to the hypotheses, we also examined whether participants' self-reports of political ideology moderated the language effect on MFQ scores. To examine this, we submitted participants' endorsement of the Individualizing and Binding indices to separate 2 (Language: relative vs. absolute) × Reasoning style (continuous) × Political ideology (continuous) regressions using the PROCESS macro (Model 3; Hayes, 2013). Results revealed that political ideology did not interact with Language to create any higher order interactions on the total score (*ps* > 0.15), Individualizing (*ps* > 0.19) or Binding subscales (*ps* > 0.36). Thus, while political ideology is associated with deontological reasoning and moral foundations, the moderating role of such reasoning on the relation between linguistic effects and agreement occurred independently of political ideology.

### Discussion

As expected, the use of absolute language led to generally less agreement on the total score of the MFQ, but only for participants low in deontological reasoning. When breaking down the MFQ by its two superordinate foundations, the predicted interaction was primarily driven by endorsement of the Individualizing subscales.

While the results are largely consistent with the hypotheses, results revealed that the Reasoning × Language interaction was driven primarily by responses to the Individualizing but not the Binding foundations. Why would participants low in deontological reasoning be more sensitive to the language effect than participants who are high in deontological reasoning but only for the individualizing foundations? One possibility is that the language effect was not as strong for the Binding foundations as it was for the Individualizing foundations. However, very similar phrases were used for both subscales, so this seems unlikely.

Another possibility is that deontological reasoning is more closely linked to the Binding foundations of loyalty, authority, and purity than the Individualizing foundations of harm and fairness, thus overpowering the language manipulation. Indeed, the correlation between the composite deontological reasoning index and the composite Binding subscale (*r* = 0.50) is greater than the reasoning index and the Individualizing subscale (*r* = 0.23), *Z* = 2.76, *p* = 0.003. The Binding foundations themselves may be more closely linked to deontological reasoning, and thus less sensitive to the language manipulation.

An additional possibility stems from the content of the scale items assessing moral reasoning. That is, the items on the CTS and CS may apply more to one set of foundations than others, and thus reasoning styles may be more sensitive to one particular set of foundations. This increased relevance to a particular set of foundations may increase the correspondence between the set and the scales, which may partly drive interaction between reasoning style and language. Examination of the topics used in the CTS reveal that the majority of the topics relate to the concept of harm – an Individualizing foundation. Specifically, 10 out of the 14 items pertain to topics or issues related to harm (e.g., killing, euthanasia, abortion, torture, betrayal, deception). We therefore believe it is more likely that the lack of the Reasoning × Language effect on endorsement of the Binding foundations are driven by the similarity of content between the moral reasoning scale and the foundations. We return to this issue later in the General Discussion.

## Study 2

Study 1 provided evidence that deontological measures of moral reasoning can moderate the effect of absolute language on the endorsement of the moral foundations. Despite this advance, a number of questions remain. One relates to the driving force behind the moderation. That is, is it the language used in the measures of moral reasoning or is it the type of reasoning being assessed by the measures? It could be that the language is driving the effects, the reasoning style, or some combination of both. To examine the question of whether the reasoning style or the language is responsible for the effects demonstrated in Study 1, in Study 2 we used a measure associated with moral reasoning that contains very little rigid or absolute language in it. Specifically, we used the Dogmatism scale (Altemeyer, 2002), a measure related to moral reasoning and morality (Rokeach, 1960). Importantly, the scale items generally do not include the absolute language style seen in previously used measures, thus removing the influence of the potential language bias. In addition, the scale does not include items specific to any moral foundations, unlike the measures used in Study 1, thus making it less sensitive to specific moral foundations. We also included the CTS used in Study 1, which will help test a conceptual replication of Study 1.

### Method

#### Participants and Procedure

Four hundred participants (172 male, 223 female; *M*_age_ = 19.52, *SD*_age_ = 2.21; 328 Caucasian, 23 Black/African American, 21 Asian/Pacific Islander, 15 Hispanic, 10 Other) recruited from a social science participant pool at a large Midwestern university participated in a 2 (Language: relative vs. absolute) × Reasoning style (continuous) design^9^^,^^10^. After consenting, participants completed the Consequentialist Thinking Style scale (CTS) used in Study 1 and the Dogmatism scale (Altemeyer, 2002). Participants were then exposed to the Language manipulation, which was embedded in the MFQ in a similar manner as in Study 1. After reporting their agreement with the MFQ items, participants reported their political ideology and demographics, were debriefed, and given course credit for participating^11^.

### Predictor Variables

#### Consequentialist Thinking Style Scale

Participants completed the same version of the CTS as in Study 1. The items were reverse scored and combined to create an overall index of reasoning style (α = 0.78). Higher scores indicate greater endorsement of deontological reasoning.

#### Dogmatism Scale

Following the CTS, participants completed the Dogmatism scale (Altemeyer, 2002), a 20-item measure that assesses the extent to which individuals hold firmly to their beliefs (e.g., *People who disagree with me are just plain wrong and often evil as well*; α = 0.85). While not directly tied to moral reasoning, participants scoring high on dogmatism tend to be more absolute and dichotomous in their reasoning style (Rokeach, 1960), which is similar to deontological reasoning. Moreover, only three of 20 items contain absolute words such as “never.” All participants reported their agreement on a 7-point scale (1 = *completely disagree*, 7 = *completely agree*). Higher scores indicate greater levels of dogmatism.

#### Political Ideology

Participants reported their political ideology on the same three items as in Study 1 (α = *0.9*1). Participants also reported their political affiliation on a 7-point scale (1 = *strong democrat*; 7 = *strong republican)*. The indices were correlated (*r* = 0.75) and were standardized and averaged to create a composite score of political ideology. Higher scores indicate greater political conservatism.

### Independent Variables

#### Linguistic Manipulation

All participants completed the same statement/agreement portion of the MFQ as in Study 1. Half of participants were provided statements with relative language whereas the other half were provided with more absolute phrases (see Appendix A for exact wording of items). Items were presented in random order.

### Dependent Measure

#### MFQ Scores

As with Study 1, participants' endorsement with the MFQ items served as the main dependent variable. All participants reported their agreement on a 6-point scale (1 = *strongly disagree*, 6 = *strongly agree*). Similar to Study 1, we averaged the Harm and Fairness measures into an index of Individualizing (α= *0.5*5) and the Loyalty, Authority, and Purity measures into an index of Binding (α= *0.8*1).

### Results

Table 4 presents the means, standard deviations, and correlations with the relevant variables. Because the CTS and Dogmatism scales were correlated, we created a composite score by standardizing the scale scores and averaging them to create a measure of deontological reasoning, which served as the moderator in the analyses. A series of 2(Language: relative vs. absolute) × Reasoning style (continuous) regressions were conducted for the MFQ total score and the Individualizing and Binding indices of the MFQ.

**Table 4:** Study 2 means, standard deviations, and correlations of the variables.

	***M* (*SD*)**	**2**.	**3**.	**4**.	**5**.	**6**.	**7**.	**8**.	**9**.
1. INDIV	5.19 (0.83)	0.34^*^	0.69^*^	−0.09	−0.10	−0.10	−0.03	−0.02	0.17^*^
2. BIND	4.67 (1.00)	–	0.92^*^	0.36^*^	0.32^*^	0.29^*^	0.14^*^	−0.10	0.03
3. MFTOT	4.88 (0.78)		–	0.24^*^	0.20^*^	0.18^*^	0.12	−0.08	0.09
4. PO	5.00 (1.99)			–	0.75	0.40^*^	0.27^*^	−0.04	0.02
5. PI	4.00 (1.64)				–	0.30^*^	0.21^*^	−0.07	−0.05
6. DOG	64.27 (17.49)					–	0.34^*^	−0.09	0.07
7. CTS	21.81 (3.97)						–	0.11^*^	−0.16^*^
8. AGE	19.52 (2.21)							–	−0.18^*^
9. GEN	–								–

* *p < 0.05. Sample size for correlations range from 394 to 399. INDIV, Individualizing foundations; BIND, Binding foundations; MFTOT, Moral foundations total scale; PO, Political orientation; PI, Political identification; DOG, Dogmatism scale; CTS, Consequentialist Thinking Style scale; AGE, Participant age; GEN, Participant gender*.

#### MFQ Total Score

Results revealed that greater deontological reasoning was associated with greater agreement on the MFQ [*b* = 0.016, *SE* = 0.05, *t*(395) = 3.52, *p* < 0.001, 95% *CI:* 0.07, 0.25]. A main effect of Language also emerged [*b* = −0.2, *SE* = 0.04, *t*(395) = −5.37, *p* < 0.001, 95% *CI:* −0.27, −0.13], with less agreement in the absolute (*M* = 4.67) than relative (*M* = 5.07) conditions. These effects were qualified by the predicted Language × Reasoning style interaction (see Table 5). Specifically, at 1 *SD* below the mean of reasoning style scores, participants reported less agreement in the absolute (*M* = 4.4) than relative (*M* = 5.07) conditions. The range of significance included 79% of the sample below the mean of the reasoning style measure. For participants 1 *SD* above the mean of reasoning style scores, the Language effect was not significant. Thus, consistent with Studies 1 and 2, participants low on deontological reasoning in the absolute conditions reported less endorsement with the moral foundation statements than in the relative conditions.

**Table 5:** Study 2 interaction effects of language × reasoning style (continuous) regression on MFQ total scores, individuating, and binding subscales.

		***b***	***SE***	***t*(395)**	***p***	**95% *CI***
MFTOT						
	Interaction	0.17	0.04	3.70	<0.001	0.08;0.25
	Decomposed at +1 *SD* Deon. reasoning	−0.06	0.05	−1.17	0.24	−0.16;0.04
	Decomposed at −1 *SD* Deon. reasoning	−0.82	0.05	−6.42	<0.001	−0.44;−0.23
INDIV subscale						
	Interaction	0.14	0.05	2.74	0.006	0.04;0.24
	Decomposed at +1 *SD* Deon. reasoning	0.13	0.06	2.14	0.03	0.01;0.24
	Decomposed at −1 *SD* Deon. reasoning	−0.1	0.06	−1.74	0.08	−0.22;0.03
BIND subscale						
	Interaction	0.19	0.06	3.35	<0.001	0.08;0.29
	Decomposed at +1 *SD* Deon. reasoning	−0.19	0.06	−2.90	0.004	−0.31;−0.06
	Decomposed at −1 *SD* Deon. reasoning	−0.49	0.06	−7.65	<0.001	−0.61;−0.36

*MFTOT, Moral foundations total scale; INDIV, Individualizing foundations; BIND, Binding foundations*.

#### Individualizing Subscale

Results on the combined Harm and Fairness subscales revealed a significant Language × Reasoning style interaction (see Table 5). Specifically, at 1 *SD* below the mean of reasoning style scores, the Language manipulation did not significantly affect agreement (absolute conditions *M* = 5.13; relative conditions *M* = 5.33), but the pattern is consistent with Study 1. The range of significance included 10% of the sample below the mean of the reasoning style measure. Interestingly, at 1 *SD* above the mean, participants in the absolute conditions reported marginally greater agreement (*M* = 5.27) than in the relative conditions (*M* = 5.02). The range of significance included 20% of the sample above the mean of the reasoning style measure. In other words, participants high on deontological reasoning reported greater endorsement of the Individualizing subscales when the items absolute language rather than less extreme language. This is inconsistent with Study 1.

#### Binding Subscale

Results on the Loyalty, Authority, and Purity subscales revealed a main effect of Reasoning style [*b* = 0.31, *SE* = 0.06, *t*(395) = 5.45, *p* < 0.001, 95% *CI:* 0.19, 0.41], with greater amounts of deontological reasoning associated with greater agreement. A main effect of Language also emerged [*b* = −0.34, *SE* = 0.05, *t*(395) = −7.47, *p* < 0.001, 95% *CI:* −0.43, −0.25], with less agreement in the absolute (*M* = 4.33) than relative (*M* = 5.0) conditions. The predicted Language × Reasoning style interaction was also significant (see Table 5). Specifically, at 1 *SD* below the mean of the reasoning style scores, participants in the absolute conditions reported less agreement (*M* = 3.92) than those in the relative (*M* = 4.9) conditions. The range of significance included 89% of the sample above the mean of the reasoning style measure. At 1 *SD* above the mean, participants in the absolute conditions also reported less agreement in the absolute (*M* = 4.73) than in the relative conditions (*M* = 5.1), but this difference was smaller than for participants low in deontological reasoning style scores The range of significance included 11% of the sample above the mean of the reasoning style measure. Put differently, participants low in deontological reasoning reported less endorsement with the Binding foundations when the subscales scale included absolute language^12^.

#### Political Ideology as a Moderator

Consistent with Study 1, the political ideology measure did not moderate the Language × Reasoning style interaction on the Individualizing (*ps* > 0.76), Binding (*ps* > 0.09) or the total score (*ps* > 0.18). We examined whether political ideology moderated the predicted interaction between idealism and language and found no evidence for such an effect^13^.

### Discussion

Consistent with Study 1, measures of deontological reasoning moderated the influence of rigid or absolute language on endorsement of moral foundations. Thus, similar to Study 1, the moderating role of reasoning on the relation between linguistic effects and agreement occurred relatively independent of political ideology.

## General Discussion

Deontological reasoning consists of rigid and absolute ways of interpreting the moral landscape (Kant, 1785/1959; Greene, 2007), and is often accompanied by the use of rigid and absolute language. This rigid language can lead to less agreement with statements of a variety of topics (Salancik and Conway, 1975) for individuals who do not view moral reasoning as so rigid. Across two studies, individuals low on deontological reasoning were less accepting of moral statements containing this rigid language.

The current studies advance work on moral reasoning by demonstrating that the language style, while likely linked to the rigid reasoning style, led to less endorsement of common moral foundations. Consistent with previous research on language and attitudes (Sherif and Hovland, 1961; Blankenship and Craig, 2011), the current studies suggest that extreme language alters the connotation of the statements, which are less likely to be endorsed by individuals low in deontological reasoning. Thus, with moral judgments, it may be more how you say it than what you say cf. (Brennan and Williams, 1995).

The current research is another example of how language influences moral reasoning. Perhaps because the association between language and cognition is relatively robust (Carroll, 1956; Zlatev and Blomberg, 2015), it should be no surprise that variations in many aspects of language can influence moral judgments and opinions. For example, individuals tend to make more utilitarian than deontological decisions when moral dilemmas are presented in a foreign language (Costa et al., 2014), and when individuals are bilingual (Wong and Ng, 2018). Our studies highlight a more subtle and interactive effect of language and thinking style on attitudes, such that the language style within a particular language (English) interacts with moral reasoning to influence agreement with rigid (deontological) statements.

One limitation of the current studies is that it is unclear whether individuals low on deontological reasoning are sensitive to extreme language more broadly, or whether it is confined to moral issues. That is, the studies do not allow for a test of extreme language in moral content vs. non-moral content. It could be that individuals low in deontological reasoning may be sensitive to absolute language in other domains beyond morality. Future work should examine extreme language effects for non-moral issues.

Another area for future research pertains to the consequences for the lowered agreement for individuals low in deontological reasoning exposed to absolute language. In particular, future research should examine whether these differences in agreement reflect differences in the moral content activated in response to the statement. For example, due to their general rejection of the statements, individuals low in deontological reasoning may generate fewer instances of moral behavior as a result of the extreme language used, compared to less rigid language.

Similarly, future research should examine whether these differences in agreement are consequential with regard to their durability and impact. For example, individuals low in deontological reasoning exposed to the absolute phrasing report less agreement with moral foundations. Does this difference translate into differences in thinking and behavior? That is, are these individuals less likely to use their opinion toward that foundation to guide their future moral thinking and behavior? Research in the attitudes and persuasion literature would suggest so (Tesser et al., 1995). In this case, moderate agreement with the absolute statements relevant to the foundation may translate into opinions toward that foundation as less likely to persist over time and be more malleable (i.e., less durable; (Krosnick and Petty, 1995) than extreme (favorable) agreement resulting from the relative items. In addition, moderate agreement in the relative conditions may also be less likely to be impactful for future thinking and behavior associated with those foundations.

An additional potentially fruitful area for future work would be to examine the role of a rigid language style in measures of moral reasoning. That is, whereas the current studies used agreement with measures of moral foundations as the outcome, one could imagine that agreement with moral reasoning measures may also be influenced by the same language style effects demonstrated in the current studies. As mentioned earlier, a number of moral reasoning scales include rigid and absolute language in their original form. It may be beneficial to compare the original measures with scale items that omit the rigid phrase or substitute it for a less rigid phrase. For example, compare the statement “*Some rules should never be broken”* from the Consequentialist Thinking scale with “*Some rules should occasionally be broken.”* Individuals with a deontological style of moral reasoning would be more likely to agree with the former statement than those with a less deontological thinking or utilitarian style. Further, these potential differences may also translate into the statement's ability to predict moral judgments. Differences in prediction as a function of language used may speak to the crucial nature of the language in moral reasoning.

The current research also highlights a broader issue associated with commonly used measures of moral reasoning. That is, many items used to assess moral reasoning seem to tap into the moral foundation of harm. Such a focus may have unintended consequences that are highlighted in the current studies. Specifically, moderation of absolute language on moral foundation endorsement occurred particularly for the Individualizing foundations, which is comprised of the Harm and Fairness foundations. This was particularly the case for scales where half or more of the items related to harm.

The current research also has implications for moral modularity debate in moral cognition (Schein and Gray, 2017). Briefly, Moral Foundations Theory assumes that the moral foundations are conceptually distinct in their outcomes and processes (i.e., modular; (Haidt, 2001). For example, violation of the purity foundation would lead to reactions relatively specific to purity (i.e., disgust) and would be distinct from reactions primarily associated with the other foundations (e.g., fairness). In contrast, the dyadic morality perspective posits that harm undergirds all moral violations (e.g., purity violations, fairness violations, etc.), and harm is the primary mechanism for these reactions to occur. Now, as mentioned earlier, measures of deontological reasoning seem to emphasize the harm foundation of morality above all others. If harm were similarly relevant to the five moral foundations examined, then the primarily harm-based deontological measure would be able to moderate the language effect on endorsement of all the foundations, and not just the individuating foundations. While this is the case for the composite MFQ measure for these two studies, the deontological measures moderated the language effect only for the individuating foundations. If harm were a primary mechanism for moral judgment as posited by the dyadic morality perspective, then one would expect that the deontological measure would moderate the language effect on endorsement for all foundations.

Why the discrepancy? A number of possibilities exist. First, it may be that the notion of harm is not as explicit in the binding foundations as it is in the individuating foundations. Indeed, research has demonstrated that violations of the various moral foundations are variable in their perceived association with harm (Schein and Gray, 2015). Thus, making harm more explicit and similarly salient may provide a stronger test of the moderating role of moral reasoning on the language effect. Second, previous research has found that the foundations of harm, fairness, and authority are associated with greater deontological reasoning (Kreps and Monin, 2014). Another possibility, albeit unlikely, is that harm is not as primal as posited by the dyadic morality perspective. Given that individuals use harm when considering what is immoral and deciding among acts which is most immoral (Schein and Gray, 2015), we do not believe that this possibility is the case. Nonetheless, future research should examine these and other possibilities.

As populations become more divided on important issues, language shifts may provide a small but important avenue for creating dialogue around the more pressing issues facing the world today. By understanding how moving from absolute to relative language we can find common starting points from which to have important conversations that keep our shared humanity at the center.

## Data Availability Statement

The raw data supporting the conclusions of this article will be made available by the authors, without undue reservation.

## Ethics Statement

The studies involving human participants were reviewed and approved by Iowa State University Institutional Review Board. The patients/participants provided their written informed consent to participate in this study.

## Author Contributions

KB and TC developed the idea, designed the studies, and wrote the manuscript with feedback and edits from MM. KB conducted and analyzed the studies with feedback from TC and MM. All authors contributed to the article and approved the submitted version.

## Conflict of Interest

The authors declare that the research was conducted in the absence of any commercial or financial relationships that could be construed as a potential conflict of interest.

## Footnotes

^1^Eight participants did not report gender, age, or race.

^2^Participants also completed part 1 of the MFQ for both studies reported.

^3^The 6-item Knowledge subscale of the epistemic belief inventory (Schraw et al., 2002) was interspersed with the MFQ to help mask the linguistic phrasing of the MFQ. These items were presented in their unaltered form.

^4^We used this condition over a control condition because we felt that without explicit relative language, participants would interpret the statements in the control condition consistent with their deontological thinking (e.g., deontological thinkers would infer that it is in fact *always* wrong to hurt a defenseless animal).

^5^While low, these reliability estimates are similar to those reported by Graham et al. (2011), even with sample sizes between 1,600 (subscale range between 0.39 and 0.70) and 28,800 (average subscale range between 0.69 and 0.86). Thus, we believe that it is more likely that the low alphas have more to do with the nature of the MFQ than the sample sizes reported in the paper and the linguistic manipulation.

^6^When the reasoning style measures were analyzed separately, the results were similar in statistical significance and pattern as the analyses reported in the main text.

^7^An alternative analysis would be to conduct a factorial Multivariate Analysis of Variance (MANOVA) with the MFQ scores as separate outcome measures. Results of the analysis was consistent with those reported in the text, such that a reasoning style × language interaction was significant for the Harm *F*_(1, 149)_ = 6.14, *p* = 0.01 and Fairness *F*_(1, 149)_ = 4.34, *p* = 0.04 scales associated with the Individuating foundations. In addition, the effect of reasoning style on the binding foundations (ps < 0.003).

^8^Because participants also completed the utilitarian scale, we conducted the same centered regression analyses with the utilitarian scale as a covariate. The results remain largely unchanged and do not affect the significance results of the analyses presented in the text.

^9^Three participants did not report gender, age, or race. An additional two did not report gender.

^10^One person did not complete the MFQ, thus reducing the sample to 399.

^11^Materials from Study 2 were part of a larger survey battery. Participants also completed part 1 of the MFQ, the Need for cognition scale (NFC; Cacioppo and Petty, 1982), and the Need for affect scale (NFA; Maio and Esses, 2001) prior to completing the demographic information. The Language manipulation was not moderated by the NFC or NFA scales.

^12^Similar to Study 1, a Language × reasoning style MAOVA with the five moral foundation measures as outcome variables. Results were consistent with the univariate analyses reported in the text, such that a reasoning style × language interaction was significant for the Harm, Ingroup, and Purity foundations (ps < 0.003) and in the expected direction for the Fairness and Authority foundations (ps < 0.16).

^13^Some readers may be wondering that, because deontological reasoning is a proxy for ideology and controlling for political ideology is attenuates or nullifies the effects. However, across both studies, centered regression analyses with political ideology as a covariate yielded no meaningful changes in the results reported.

## Appendix

**Appendix A**. MFQ items used with language manipulation.

**Relative Language Condition**

1. Sometimes, compassion for those who are suffering should be a virtue.
2. When making laws, the government should at times ensure that the law treats people fairly.
3. Occasionally, I am proud of my country's history.
4. Respect for authority is probably something children should learn at some point.
5. People should probably not do things that are disgusting, even if no one is harmed.
6. Sometimes it can be wrong to hurt a defenseless animal.
7. Justice is at times an important requirement for a society.
8. People should at times be loyal to their family members, even when they have done something wrong.
9. At times, men and women will have different roles to play in society.
10. I would sometimes call some acts wrong on the grounds that they are unnatural.
11. It is sometimes right to kill a human being.
12. There are times where I think it's morally wrong that rich children inherit a lot of money while poor children inherit nothing.
13. Occasionally, it is more important to be a team player than to express oneself.
14. If I were a soldier and disagreed with my commanding officer's orders, I might sometimes obey anyway because that is my duty.
15. Sometimes, chastity can be an important and valuable virtue.

**Absolute Language Condition**

1. Compassion for those who are suffering should forever be a virtue.
2. When making laws, the government should always ensure that the law treats people fairly.
3. I am constantly proud of my country's history.
4. Respect for authority is definitely something all children need to learn.
5. People should never do things that are disgusting, even if no one is harmed.
6. It is always wrong to hurt a defenseless animal.
7. Justice is always an important requirement for a society.
8. People should always be loyal to their family members, even when they have done something wrong.
9. Men and women will always have different roles to play in society.
10. I would call some acts as always wrong on the grounds that they are unnatural.
11. It is never right to kill a human being.
12. I think it's always morally wrong that rich children inherit a lot of money while poor children inherit nothing.
13. It is always more important to be a team player than to express oneself.
14. If I were a soldier and disagreed with my commanding officer's orders, I would always obey anyway because that is my duty.
15. Chastity will always be an important and valuable virtue.
